# A Psychometric Study of the Bayley Scales of Infant and Toddler Development in Persian Language Children

**Published:** 2017

**Authors:** Nadia AZARI, Farin SOLEIMANI, Roshanak VAMEGHI, Firoozeh SAJEDI, Soheila SHAHSHAHANI, Hossein KARIMI, Adis KRASKIAN, Amin SHAHROKHI, Robab TEYMOURI, Masoud GHARIB

**Affiliations:** 1 Pediatric Neurorehabilitation Research Center, University of Social Welfare and Rehabilitation Sciences, Tehran, Iran; 2 Department of Psychology, Karaj Branch, Islamic Azad University, Karaj, Iran; 3 Ph.D Candidate, Faculty of Paramedicine, Mazandaran University of Medical Sciences, Sari, Iran

**Keywords:** Child Development, Psychometery, Bayley

## Abstract

**Objective:**

Bayley Scales of infant & toddler development is a well-known diagnostic developmental assessment tool for children aged 1–42 months. Our aim was investigating the validity & reliability of this scale in Persian speaking children.

**Materials & Methods:**

The method was descriptive-analytic. Translation- back translation and cultural adaptation was done. Content & face validity of translated scale was determined by experts’ opinions. Overall, 403 children aged 1 to 42 months were recruited from health centers of Tehran, during years of 2013-2014 for developmental assessment in cognitive, communicative (receptive & expressive) and motor (fine & gross) domains. Reliability of scale was calculated through three methods; internal consistency using Cronbach’s alpha coefficient, test-retest and interrater methods. Construct validity was calculated using factor analysis and comparison of the mean scores methods.

**Results:**

Cultural and linguistic changes were made in items of all domains especially on communication subscale. Content and face validity of the test were approved by experts’ opinions. Cronbach’s alpha coefficient was above 0.74 in all domains. Pearson correlation coefficient in various domains, were ≥ 0.982 in test retest method, and ≥0.993 in inter-rater method. Construct validity of the test was approved by factor analysis. Moreover, the mean scores for the different age groups were compared and statistically significant differences were observed between mean scores of different age groups, that confirms validity of the test.

**Conclusion:**

The Bayley Scales of Infant and Toddler Development is a valid and reliable tool for child developmental assessment in Persian language children.

## Introduction

Developmental disabilities are common pediatric problems. The prevalence of children with developmental delays is about 12% to 16% of the general pediatric population (1). In developed countries in 2006 –2008, one out of every six children had developmental difficulties. The prevalence of developmental disabilities had increasing trends over past 12 years (from 12.84% to15.04%) (1).The number of children with specific developmental disabilities such as autism and attention deficit hyperactivity disorder has been increased (1). 

The prevalence of global developmental delay in Iranian children is estimated to be 14.6% (2). At least 3.7% of children under 18 months have motor impairment (3-5). There is little data on the prevalence of developmental disorders in children below 3 yr because of limited availability of reliable and valid instruments that can be used to assess young children in large surveys (6). On the other hand, some studies support the effectiveness of early intervention in children with developmental problems (7, 8). Besides, “timely and effective interventions for neurodevelopmental disorders can lead to more parental assurance and give pediatricians more confidence in referring families for early intervention services”(9). Therefore, early detection of developmental disorders is critical for the health of children and families and is a major task for primary care pediatricians (10). Screening is a brief assessment way to find children in need of more accurate diagnostic evaluation (11). 

Despite increased evidence regarding the importance of development and growth of young children, formal screening for identifying developmental and behavioral difficulties is often not included in general pediatric practice (12). 

Bayley scales of infants & toddlers development (Bayley) is an individually administered instrument that evaluates the state of development in children 1 to 42 months in cognitive, communication (receptive & expressive), and motor (gross & fine) domains. Bayley scale can be used for early detection of developmental disorders, such as motor, language and cognitive disorders (13). 

In this study we aimed to investigate the reliability and validity of the Bayley scales in Persian language children.

## Materials & Methods

This research was a cross-cultural psychometric study of the Bayley scales of infant & toddler development, for 1- 42 months children .The method was descriptive – analytic. 

The Persian version of test was developed through precise translation and back-translation as well as cultural adaptation. Translation and back translation of test were done by research team consisted of 5 pediatricians; 2 occupational therapists and 1 linguist, expert in early child development and also familiar with English language. Comparing results of translation and back translations, discrepancies were extracted and corrected. The result was then assessed for face and content validity by experts consisting of 9 faculty members with occupational therapy, speech language pathology, psychology and pediatrics specialties. Using comments and opinions from these experts, cultural and language adaptations were performed. Because of major differences between Persian and English literature, changes were done predominantly on communication subtests. Besides, some photos in stimulus and picture book were changed.

**Participants**

Overall, 403 children 1- 42 months in 17 age group (A to Q) were examined by the Bayley. Children were recruited from health care centers of Tehran, Iran in 2013- 2014. 

The inclusion criteria were 1) age range 1 to 42 months; 2) apparently normal development & lacking any apparent developmental disorders; 3) Persian language; Written informed consent was taken from parents. Bayley was administered by examiners who had Master degree of Occupational Therapy or Psychology trained for test administration.

**Psychometric properties of the scale**

**Reliability**

Reliability was estimated through calculating Cronbach’s alpha coefficient for internal consistency of items separately in all five subtests. 

For determining test- retest reliability, 45 children were re- tested by same examiner within 4-7 days after first administration. 

In addition, 36 children were rescored by another examiner in order to determine inter- rater reliability.

**Validity**

Face and content validity were investigated by experts’ opinions. 

Construct validity was determined by two ways: factor analysis and comparing mean raw scores in all 17 age groups.

**Factor analysis**

First KMO ^1^ values were calculated as 0.942-0.964 for different domains; this indicated sample adequacy for performing factor analysis. Then Bartlett’s test of sphericity was done and Chi square was calculated as 202553/901 with a significance of P < 0.0001.This indicated that correlation matrices between test items did not equal zero. Therefore, performing the factor analysis based on correlation matrices between items of the test was explicable. Then, factor analysis was done using the Principal Components (PC) analysis method. In order to determine that test components are saturated by how many significant factors, three determinants were considered: special value; relative variance expressed by each factor; and special value or scree plot.

**Comparing mean raw scores**

Since the nature and content of the test was based on child development during time; so it was assumed that the function of participants should have a relationship with chronological age. Consequently, for testing this hypothesis, the raw score of each subtest in different age groups were compared by means of one-way variance analysis.

**Analysis**

Descriptive statistical methods were used to describe the participants. Internal consistency was quantified by means of Cronbach’s alpha. The test–retest and Interrater reliability were quantified by means of the ICC, using Pearson’s non-parametric coefficient. The level of significance was defined as P<0.01. SPSS version 16 (Chicago, IL, USA) was used for data analysis.

## Results

**Precipitants**

Of 403 children, 51.6% were boys. Children were from 1 to 42 months ages in 17 age groups. The minimum number of children (n=10) were in age group E (4 mo & 16 days- 5 mo & 15 days); and maximum number (n=38) were in age group D (3 mo & 16 days- 4 mo & 15 days).

**Reliability**

Mean Alpha Cronbach values for internal consistency in different developmental domains are shown in Table I. The lowest alpha Cronbach value belonged to receptive communication and highest to cognitive domains. 

The test- retest reliability values obtained in terms of Pearson correlation coefficient were 0.982 for receptive communication; 0.983 for fine motor; 0.987 for expressive communication; 0.991 for cognitive; and 0.995 for gross motor domains. The level of significance was P< 0.01. 

The inter- rater reliability values obtained in terms of Pearson correlation coefficient were, 0.993 for fine motor; 0.996 for expressive communication; 0.995 for both receptive communication and gross motor; and 0.997 for cognitive domains. 

**Validity**

Face and content validity of test were approved by experts’ opinions. Construct validity was determined by two ways: factor analysis and comparing mean raw scores.

**Factor analysis**

The goal of performing factor analysis for all subtests was extraction of one factor from each subtest items; so the special value and percentage of variance expressed by the first factor were calculated and it was concluded that the best condition for performing factor analysis in items of each subtest was a one-factor model. 

In addition, scree plot of the test showed that contribution of first factor in total variance of each subtest was significant and distinct. Therefore, from all items of each subtest, one factor was extracted. 

In order to investigate on the nature of relationships between test items, and to reach the definition of factors, it was assumed that coefficients above 0.3 were significant and consequently coefficients below 0.3 were considered accidental.

**Comparing mean raw scores**

The results indicated that a correlation existed between age and test scores in all five dimensions such that an increase in age resulted in an increase in score in each of the five dimensions.

## Discussion

The present study intended to determine the validity and reliability of the Bayley scales of infant & toddler development Persian language children. Our findings have confirmed the validity and reliability of Persian version of Bayley Scales. 

**Table 1:** Internal Consistency of Bayley Scales of Infant & Toddler Development

	**Cognitive**	**Receptive communication**	**Expressive communication**	**Fine motor**	**Gross motor**
Mean alpha crohnbach	0.84	0.74	0.82	0.82	0.82

The world health organization (WHO) has prioritized early detection of developmental disorders in children (14) as early interventions are keys to minimizing long-term impacts of developmental delays (15). 

Therefore, employing standard tools in early detection of developmental delays in children is of paramount importance. Besides, the same tools are used in evaluating the efficiency of interventions (16). 

The Health Care System of Iran needs a standardized child development evaluation test; there were no standard developmental test for precise detection of developmental delay in Iranian children suspected to have developmental delay during a screening test. 

Furthermore, studies regarding child development screening tools in IRAN showed the need for a standard diagnostic test for comparing the results of screening tools and determining concurrent validity of screening tests with a gold standard (17, 18). 

The existing tools in Western countries are only applicable for their own context as using them in other countries would pose limitations in interpreting the scores (19). Thus, it is necessary to reevaluate the reliability and validity of such instruments and apply appropriate cultural adjustments. 

Bayley scales of infant & toddler development is an individually administered developmental test which evaluates child development in areas of cognition, communication, and motor. It has been designed for children between the ages of 1 - 42 months. 

The original Bayley was designed and normalized in the U.S considered as a gold standard for assessing infant development (20). In addition, test-retest reliability of original Bayley was calculated by administering the Cognitive, Language and Motor Scales twice to a group of 197 children. Reliability coefficients ranged between 0.67-0.94, with correlations increasing as age increased (21). 

Evidence for internal consistency of original test and the average reliability coefficients for the subtests were: cognitive 0.91, receptive communication 0.87, expressive communication 0.91, fine motor 0.86, and gross motor 0.91(21). Accordingly, we decided to provide Persian version of Bayley scale and determine its validity and reliability. In our study, alpha Cronbach coefficient in all domains was above 0.74, which shows very good internal consistency between items of subscales. 

For reliability determination using test- retest administration, in all subscales, Pearson Correlation coefficient was ≥0.982 (P< 0.01); which shows excellent correlation between two administrations. Besides, interrater reliability using Pearson Correlation coefficient was ≥0.993 (P<0.01) in all subscales; that means excellent correlation between examiners. 

The results obtained from confirmatory factor analysis indicated that Bayley Scales is valid for evaluating infant development aged 1-42 months in Tehran. Additionally, comparison of means showed that the mean values in different age groups were significantly different. A correlation existed between age and test scores in all five dimensions such that an increase in age resulted in an increase in score in each of the five dimensions. These findings confirmed the hypothesis that test performance was associated with age and thus the validity of this test was confirmed once again. 

The results of the current study confirmed the findings of Godamunne et al. who reported a very high reliability for Bayley Scales through test-retest (22). Furthermore, our results are aligned with another study, which evaluated the reliability of Bayley Scales using interrater agreement between two trained raters. That study reported very high levels of interrater reliability for Bayley Scales (23). 

The high reliability of Malay Version of Bayley Scales was reported through examining internal consistency and calculating Cronbach’s alpha (24). The authors used back to back translation to provide Malay translation of the scale. The methods and results of that study are aligned with the present research, too. They also evaluated the convergent validity of the five subscales and the results indicated that there was a high positive correlation between them (24). 

The findings of the present study are also aligned with research results of Yu et al. whose research showed good to excellent reliability for Bayley Scales using testretest and interrater reliability (25). They confirmed the validity of the test by employing concurrent validity and correlation between this test and its second edition. They claimed that Bayley Scales-third edition evaluated the development levels of Taiwanese children higher than the second edition. Therefore, they suggested raising the cut-off points in the third edition so that it would show developmental delays in children (25). 

Other studies have also pointed out this issue, which mostly affects cognitive domain of the test; that is, evaluating a child’s developmental level with Bayley Scales-third edition might overestimate child’s development or underestimate developmental disorders (26, 27). This topic can be further investigated by Iranian researchers. 

Campbell et al., reported weak to moderate correlation between different domains of Bayley Scales and Test of Infant Motor Performance (TIMP) by evaluating convergent and divergent validity of Bayley test and TIMP. They concluded that TIMP was more appropriate for early evaluation of infants (28). 

In view of limitations of the Persian developmental diagnostic tests, the present study did not employ convergent validity (simultaneous comparison of two test results). Hence, it is recommended that other researchers investigate the construct validity of the test through this method. 

Determination of developmental levels of children with special needs is a challenge that Bayley Scales has overcome by applying particular considerations. Visser et al. translated Bayley Scales into Dutch in order to provide an appropriate assessment tool for evaluating development of children with special needs. They evaluated the fitness and validity of the adapted edition for children with special needs and the results indicated that the applied adaptations have improved the validity of the test in children who had mild to moderate vision impairment and motor skills disorder. The increase in validity was more evident in the cognitive domain (29). 

The Bayley Scales has an additional screening test, which is a short form of its diagnostic edition used for screening developmental delays. Soleimani et al. have evaluated psychometric properties of the screening version of Bayley Scales and confirmed the validity and reliability of it for Persian speaking children (30). 

The diagnostic version of Bayley Scale has some limitations, too; such as the time it takes and its relatively high cost; that the screening version does not have. 

In conclusion, the Bayley Scales meets acceptable standards in reliability and validity, thus it is possible to use it for Persian-speaking children aged 1-42 months. We expect that this test fill the need for having a standardized measure for a more precise developmental assessment for children, suspected to have developmental disorders during screening tests, and hope that this test will fill the void for a gold standard test that can be used to compare with other developmental tests.
